# Family Perceptions of Quality of End-of-Life Care in Stroke

**DOI:** 10.1089/pmr.2020.0041

**Published:** 2020-07-23

**Authors:** Netana Markovitz, Lewis B. Morgenstern, Fatema Shafie-Khorassani, Bridget A. Cornett, Sehee Kim, Carmen Ortiz, Rebecca J. Lank, Erin Case, Darin B. Zahuranec

**Affiliations:** ^1^ University of Michigan Medical School, Ann Arbor, Michigan, USA.; ^2^ Stroke Program, Department of Neurology, University of Michigan Medical School, Ann Arbor, Michigan, USA.; ^3^ Department of Epidemiology and University of Michigan School of Public Health, Ann Arbor, Michigan, USA.; ^4^ Department of Biostatistics, University of Michigan School of Public Health, Ann Arbor, Michigan, USA.; ^5^ Center for Bioethics and Social Sciences in Medicine, Michigan Medicine, Ann Arbor, Michigan, USA.

**Keywords:** palliative care, quality of health care, stroke, surrogate decision makers

## Abstract

***Background:*** Most end-of-life decisions after stroke are made by a surrogate decision maker, yet there has been limited study of surrogate assessment of the quality of end-of-life stroke care.

***Objective:*** To assess surrogate perceptions of quality of end-of-life care (QEOLC) in stroke and explore factors associated with quality.

***Design:*** Cross-sectional analysis of interviewer-administered survey.

***Settings/subjects:*** Surrogate decision makers for deceased stroke patients in a population-based study.

***Measurements:*** The primary outcome was the validated 10-item family version of the QEOLC scale. The univariate association between prespecified patient and surrogate factors and dichotomized QEOLC score (high: 8–10, low: 0–7) was explored with logistic regression fit using generalized estimating equations.

***Results:*** Seventy-nine surrogates for 66 deceased stroke cases were enrolled (median patient age: 76, female patient: 53%, Mexican American patient: 59%, median time from stroke to death: seven days, median surrogate age: 59, and female surrogate: 72%). The overall QEOLC was generally high (median 8.3, quartiles 6.1, 9.6) although several individual items had a high proportion (∼30%–50%) of surrogates who felt that the questions did not apply to the patient's situation. No hypothesized factors were associated with QEOLC score, including demographics, stroke type, location/timing of death, advance directives, health literacy, or understanding of patient wishes.

***Conclusions:*** Surrogates reported generally high QEOLC. Although this finding is encouraging, modifications to the QEOLC may be needed in stroke as some surrogates were unable to provide a valid response for certain items.

## Introduction

Stroke is the fifth leading cause of death in the United States.^1^ Most early deaths after stroke occur after a decision to limit the intensity of treatment rather than from the direct effects of the stroke.^2^ Owing to neurological impairment, these decisions on life-sustaining treatments are typically made by a surrogate decision maker, most commonly a family member. The sudden onset and steeper trajectory of decline after acute stroke varies from other common illnesses such as cancer and dementia,^3^ although there has been limited study of surrogate perspectives on the quality of end-of-life care (QEOLC) after stroke.^4^^,^^5^ High QEOLC has been defined as patient-centered care that includes open and honest communication, skillful response to emotional needs, and effective symptom management.^6^^,^^7^

We report preliminary findings from a population-based stroke study focused on family surrogate decision makers' perceptions of the quality of death and end-of-life care after stroke. The objectives were to describe the overall QEOLC in stroke and explore patient or surrogate-level predictors.

## Methods

### Study participants and recruitment

Participants were recruited from the Brain Attack Surveillance in Corpus Christi (BASIC) and the companion Outcomes Among Surrogate decision makers In Stroke (OASIS) studies from April 2016 to July 2018. BASIC is a population-based stroke surveillance study in the community of Nueces County on the Texas Gulf Coast.

Details of BASIC have been reported previously^8^; in brief, active and passive surveillance are used to identify cases of ischemic stroke and nontraumatic intracerebral hemorrhage. OASIS is a substudy of BASIC focused on stroke surrogate decision makers. BASIC participants with stroke are screened for surrogate decision makers who self-identify as involved in conversations with the health care team about one of five treatment decisions: do-not-resuscitate orders, feeding tube, mechanical ventilation, brain surgery, or consideration of palliative care/comfort care/hospice.

Up to five surrogates per patient can be enrolled, and contact is deferred for a minimum of four weeks postdeath as is standard for bereaved family research.^9^ This study included BASIC and OASIS participants with at least one surrogate completing a postdeath questionnaire.

### Data collection and measures

Participants completed an interviewer-administered survey either in-person or by telephone. All surveys were available in English or Spanish and were conducted in the participant's preferred language (only one interview was conducted in Spanish).

The primary outcome was the validated QEOLC scale.^7^^,^^10^ We used the 10-item family version of the QEOLC, scored on a 0–10 scale where higher scores indicate better ratings. The original self-administered QEOLC included a choice of “does not apply/NA.” We also included a “don't know” option to maintain consistency with other study questionnaires. Participants were only offered “NA” (not ascertained) or “don't know” if they were struggling to select a response. As a secondary outcome, we assessed three selected items from the quality of death and dying (QODD) scale^11^ that addressed the overall quality of care and quality of dying.

Other surrogate characteristics assessed included self-reported history of surrogate depression or anxiety,^12^ confidence in ability to serve as a surrogate,^13^ and health literacy.^14^ We also assessed presence of patient advance directives and prior informal discussions about advance care planning. Surrogates reported on whether patients were able to participate in any of the five medical decisions mentioned earlier. Trained abstractors reviewed the medical record for additional key details, including receipt of hospice or palliative care services, or transition to comfort-measures-only.

### Statistical analysis

An overall QEOLC score was computed as an average of completed items (i.e., don't know or NA responses were removed from a respondent's average score). As surrogates were clustered within patients, we calculated the interclass correlation (ICC) as the ratio of between-patient variation over the total variation for each item with variance components estimated using a random effects model with a patient-specific random intercept. Responses to individual QEOLC and QODD items as well as the overall scores were dichotomized into low (0–7) or high (8–10) as responses were highly left-skewed.

The univariate association between prespecified patient and surrogate factors and the dichotomized overall QEOLC score were explored with logistic regression models fit using generalized estimating equations to account for multiple surrogates per patient. Multivariable adjustment was not performed due to the small sample size.

As a sensitivity analysis, we investigated the association between ordinal QEOLC (five-levels: 0–2.99, 3–4.99, 5–6.99, 7–8.99, and 9–10) and potential predictive factors using the Cochran–Armitage trend test (categorical) or correlations (continuous).

The study was approved by the Institutional Review Boards (IRBs) of the University of Michigan and the local hospitals. All surrogates signed informed consent or completed an IRB-approved telephone consent. Consent was obtained from all interviewed patients or their proxy.

## Results

From April 2016 to July 2018, 244 patients were identified as OASIS eligible and 165 (68%) had at least one surrogate agree to participate. There was no significant difference in participants versus nonparticipants by patient age, race/ethnicity, gender, or stroke type. A total of 66 deceased patients and 79 surrogates were included.

Surrogate and patient characteristics are summarized in Tables 1 and 2. Surrogates were most commonly female, Mexican American, and the patient's children. Less than half of surrogates reported that the patient had a formal advance directive, but most reported that they had had prior discussion of treatment preferences with the patient.

**Table 1.** Surrogate Descriptive Characteristics and Association with QEOLC (*n* = 79)

Characteristics	Overall median (IQR) or column %	High QEOLC (8–10), median (IQR) or n	Low QEOLC (0–7), median (IQR) or n	OR^a^ (95% CI)	p
Age (years)	59 (50, 66)	60.5 (47.5, 66.0)	58 (52.0, 64.0)	1.00 (1.00–1.03)	0.89
Gender					
Female	72.2	33	24	1.38 (0.57–3.33)	0.48
Male	27.9	11	11	ref	
Race/ethnicity					
Mexican American	58.2	25	21	1.04 (0.43–2.54)	0.93
Non-Hispanic White	38.0	16	14	ref	
Other^b^	3.8	3	0	–	
Relationship					
Spouse of patient	22.8	10	8	ref	ref
Child of patient	65.8	31	21	1.18 (0.41–3.39)	0.76
Other	11.4	3	6	0.40 (0.07–2.21)	0.29
History of depression^c,d^					
Yes	26.6	9	12	0.47 (0.16–1.38)	0.17
No	72.2	35	22	ref	ref
History of anxiety^e^					
Yes	26.6	12	9	1.08 (0.39–3.00)	0.88
No	73.4	32	26	ref	ref
Informal discussions about advance care planning^f^					
Yes	88.6	39	31	1.01 (0.25–4.06)	0.99
No	11.4	5	4	ref	ref
Formal advance directive^g^					
Yes	38.0	17	13	1.01 (0.43–2.64)	0.89
No	62.0	27	22	ref	ref
Self-reported confidence in ability to serve as surrogate^c,h^					
High	82.3	38	27	2.25 (0.67–7.61)	0.19
Low	16.5	5	8	ref	ref
Health literacy^i^					
High	78.5	36	26	1.56 (0.50–4.85)	0.44
Low	21.5	8	9	ref	ref
Time from death to interview (days)	61 (42, 85)	64 (47.5, 89)	53 (40, 85)	1.00 (0.99–1.01)	0.88

^a^ OR >1 indicates a positive association with high quality of care, whereas an OR <1 indicates a negative association.

^b^ Three identified as “other” were excluded from the model due to zero counts.

^c^ One missing value.

^d^ Defined as: “have you ever been told by a doctor or other health professional that you have depression”?^11^

^e^ Defined as: “have you ever been told by a doctor or other health professional that you have anxiety”?^11^

^f^ Informal discussions about advance care planning was assessed by asking: “have you ever discussed with [patient] the treatments [he/she] would want (or would not want) if he/she were too sick to speak for him/herself?^12^

^g^ Presence of patient advance directives was assessed by asking surrogates: “before the stroke did [patient] have an advance directive, living will, or durable power of attorney for health care?” Advance directives were defined for surrogates as “written documents that describe who will make treatment decisions and what kinds of treatment should be performed.”

^h^ Self-reported confidence in ability to serve as a surrogate was assessed by asking: “how well do you think you understand the treatments [patient] would want or would not want in his/her current medical situation?”^12^ Responses were assessed on a 0–10 scale with high confidence defined by a score of 8 or above based on the visual inspection of the response distribution.

^i^ Health literacy was assessed with a single item: “how confident are you filling out medical forms by yourself?” with responses of “extremely” or “quite a bit” considered high health literacy.^13^

CI, confidence interval; IQR, interquartile range; OR, odds ratio; QEOLC, quality of end-of-life care.

**Table 2.** Patient Descriptive Characteristics and Association with QEOLC (*n* = 66)

Characteristics	Overall median (IQR) or column %	High QEOLC (8–10), median (IQR) or n	Low QEOLC (0–7), median (IQR) or n	OR^a^ (95% CI)	p
Age (years)	75.5 (65, 88)	76 (69, 87)	73 (61, 90)	1.02 (0.99–1.05)	0.28
Gender					
Female	53.0	21	14	1.56 (0.67–3.66)	0.30
Male	47.0	14	17	ref	
Race/ethnicity					
Mexican American	59.1	21	18	0.92 (0.38–2.26)	0.86
Non-Hispanic White	37.9	12	13	ref	ref
Other^b^	3.0	2	0	-	-
Stroke type					
Intracerebral hemorrhage	27.3	7	11	0.49 (0.20–1.21)	0.12
Ischemic stroke	72.7	28	20		
Stroke Severity—National Institutes of Health Stroke Scale	18 (9, 27)	18 (9, 25)	20 (9, 30)	0.97 (0.94–1.00)	0.07
Palliative care or Hospice^c^					
Yes	40.9	14	13	0.92 (0.38–2.20)	0.85
No	53.0	17	18		
Location of death					
Hospital	51.5	18	16	ref	ref
Inpatient Hospice	31.8	12	9	1.21 (0.49–3.00)	0.67
Home	13.6	5	4	1.01 (0.24–4.35)	0.99
Other nursing facility^b^	3.0	0	2	-	-
Patient participation in decisions^d^					
Yes	15.2	5	5	0.95 (0.29–3.05)	0.93
No	84.9	30	26	ref	
Time to comfort care (days)^e^	3 (1, 5)	3 (1, 5)	3 (1, 7)	0.97 (0.88–1.07)	0.57
Time to death (days)	7 (3, 13)	6 (3, 12)	8 (3, 15)	1.01 (0.99–1.03)	0.36

^a^ OR >1 indicates a positive direction of association with high quality of care, whereas an OR <1 indicates a negative association.

^b^ Excluded from the model due to low counts.

^c^ Based on medical record review indicating any involvement of hospice or palliative care services; four missing values.

^d^ Each surrogate was asked a series of questions of the form “How much was [patient] able to participate in any conversations about [treatment]?” for up to five treatment decisions (do-not-resuscitate, feeding tube, mechanical ventilation, brain surgery, or consideration of palliative care/comfort care/hospice). Response options were “Not at all,” “Only a little,” “Some,” and “A lot.” This was collapsed and dichotomized by patient as a “yes” if any surrogate indicated “Some” or “A lot” for at least one treatment decision, and “No” otherwise.

^e^ Among the 51 patients who transitioned to comfort care.

The overall QEOLC was generally high (median 8.3, quartiles 6.1, 9.6). Table 3 describes surrogate ratings of individual items from the QEOLC and QODD. Surrogates were not able to provide a numerical analyzable response for several QEOLC items (4 of 10 items had >25% of surrogates respond “NA” or “don't know”). The ICC, which assesses the degree of agreement among different surrogates for the same patient, ranged from 0 (no correlation among surrogate responses) to 0.83 as shown in Table 3.

**Table 3.** Individual Item Responses Assessing Quality of Death and End-of-Life Care

Item question	Low score (0–7), %	High score (8–10), %	Don't know or NA (%)	ICC^a^
QEOLC				
Talks to patient in honest way	19	28	53	0.16
Responsive to patient's emotional needs	28	38	34	0.52
Helps patient get consistent information from health care team	41	53	6	0.36
Takes patient wishes into account when treating pain, symptoms	22	63	15	0.50
Admits when s/he does not know	23	38	39	0.14
Treats whole person, not just disease	30	66	4	0.36
Knowledgeable about care needs during dying process	25	71	4	0.11
Openly and willingly communicates with you	25	72	3	0
Acknowledges and respects patient and family beliefs	23	54	23	0.72
Makes patient feel comfortable s/he will not be abandoned before death	25	37	38	0.28
Overall proportion of average QEOLC score	44	56	0	0.40
QODD				
Rate the care patient received from all doctors and other health care providers (including nurses, caseworkers, and other health care professionals) during the last several days of his or her life while in the hospital	20	68	11	0.74
Rate the care patient received from his or her doctor during the last several days of his or her life while in the hospital	15	65	20	0.83
Overall, how would you rate the quality of patient's dying?	20	62	18	0.78
Overall proportion of average QODD score	19	70	11	0.82

^a^ Items with higher ICC indicates greater agreement among family members. ICC = 0 indicates no correlation among surrogates' responses.

ICC, interclass correlation; NA, not ascertained; QODD, quality of death and dying.

The associations between surrogate or patient factors with QEOLC are shown in Tables 1 and 2. None of the investigated patient or surrogate factors were associated with QEOLC. The sensitivity analysis investigating ordinal QEOLC found no association with patient or surrogate factors other than NIHSS, with more severe strokes associated with lower QEOLC (correlation coefficient = −0.25, *p* = 0.03).

## Discussion

We found that surrogate ratings of QEOLC using the validated family version of the QEOLC and selected QODD items were generally high.^15,16^ We found no patient or surrogate factors to be associated with QEOLC. The generally high rating of QEOLC across patient and surrogate subgroups is encouraging and suggests that most surrogates are satisfied with the end-of-life care their loved one received.

Although we found generally high QEOLC scores, prior study in stroke populations has identified several opportunities for improvement in the end-of-life care of stroke patients and families.^5,17^ Specific areas for improvement in end-of-life stroke have included symptom control and communication. It is possible that these important domains were not adequately assessed by the 10-item version of the QEOLC administered: there was limited assessment of symptom control in the included QEOLC items, and one of the primary items assessing physician communication (“talks to [patient] in an honest and straightforward way”) had more than half of participants respond “don't know” or “NA.” Future studies of end-of-life stroke care may wish to include additional scales more specifically focused on symptom control and communication.

In addition to the item assessing physician communication listed earlier, there were several other QEOLC items with high proportions of “don't know” or “NA” responses, which were ultimately excluded from the final score calculation.^7,10^ Although we did not conduct any formal factor analysis, these responses seemed to be more common when addressing interaction with the patient themselves, as opposed to the surrogate (e.g., “talks to [patient] in an honest and straightforward way” and “makes [patient] feel confident that he/she will not be abandoned prior to death.”) Only 15% of our patient sample was able to participate in treatment decisions, suggesting that most were unable to communicate with physicians. Thus, surrogates may have deemed these questions on communication with the patient irrelevant. Based on the proportion of items with nonanalyzable data, the 10-item version of the QEOLC may not be the optimal scale for use in stroke patients or other populations with severe neurological injury who are unable to communicate. At a minimum, researchers who use the QEOLC in the future may wish to include a formal assessment of the patient's ability to communicate over the course of treatment to explore how this scale may be affected by communication.

The sensitivity analysis suggested an association between more severe initial stroke severity and lower QEOLC, although this finding should be interpreted with caution due to the lack of multivariable adjustment, and the fact that the sensitivity analysis did not account for the correlation between respondents for a given patient. However, it is possible that patients with more severe strokes have greater palliative care needs or more surrogate distress or dissatisfaction with care, leading to lower ratings on QEOLC. Given the limitations in our data, this finding should be confirmed in other data sets.

Other factors that have previously been associated with quality of end-of life care in nonstroke populations have included race, duration of hospital stay, or certain aspects of the physician–patient relationship.^16,18,19^ Recently, the potential impact of the expanded time window for advanced stroke treatment on goal-concordant care has been reviewed.^20^ Most patients in this study were enrolled before the expansion of the endovascular treatment time window to 24 hours and, therefore, it is unlikely that these considerations impacted our findings.

Our study had limitations, including our small sample size and inability to conduct multivariable adjustment, although there are relatively few other studies examining the QEOLC in stroke. Our desire to minimize respondent burden and use abbreviated scales (e.g., 10-item QEOLC rather than the 22-item scale, and 3 selected items from the QODD) limited our ability to more fully assess important domains of the QEOLC in stroke. In addition, we were only able to assess data from surrogates and do not have a true assessment of patient treatment goals and preferences. As surrogate prediction of patient treatment preferences may be inaccurate in up to one third of cases,^21^ our findings may not be truly reflective of patient preferences. Future study should focus on developing or adapting palliative and end-of-life assessments that are appropriate for proxy completion in patients with severe neurological injury and inability to communicate.^22^ Finally, although we assessed surrogate history of depression or anxiety, we did not assess ongoing distressing symptoms such as grief and suffering, which may have negatively impacted ratings of end-of-life care.

## Conclusions

In summary, we found relatively high ratings of QEOLC among stroke surrogate decisions makers. Further study in larger populations, with particular attention to selection of the optimal scales to assess symptom control and the QEOLC in stroke patients, is warranted.

## Abbreviations Used

BASIC: Brain Attack Surveillance in Corpus Christi
CI: confidence interval
ICC: interclass correlation
IQR: interquartile range
NA: not ascertained
NIHSS: National Institutes of Health Stroke Scale
OASIS: Outcomes Among Surrogate decision makers In Stroke
OR: odds ratio
QEOLC: quality of end-of-life care
QODD: quality of death and dying
